# Early social isolation impairs development, mate choice and grouping behaviour of predatory mites

**DOI:** 10.1016/j.anbehav.2017.02.024

**Published:** 2017-05

**Authors:** Peter Schausberger, Marian Gratzer, Markus A. Strodl

**Affiliations:** a Department of Behavioural Biology, University of Vienna, Vienna, Austria; b Group of Arthropod Ecology and Behavior, Department of Crop Sciences, University of Natural Resources and Life Sciences, Vienna, Austria

**Keywords:** group living, mite, sociability, social deprivation, social enrichment, stress

## Abstract

The social environment early in life is a key determinant of developmental, physiological and behavioural trajectories across vertebrate and invertebrate animals. One crucial variable is the presence/absence of conspecifics. For animals usually reared in groups, social isolation after birth or hatching can be a highly stressful circumstance, with potentially long-lasting consequences. Here, we assessed the effects of social deprivation (isolation) early in life, that is, absence of conspecifics, versus social enrichment, that is, presence of conspecifics, on developmental time, body size at maturity, mating behaviour and group-living in the plant-inhabiting predatory mite *Phytoseiulus persimilis*. Socially deprived protonymphs developed more slowly and were less socially competent in grouping behaviour than socially enriched protonymphs. Compromised social competence in grouping behaviour was evident in decreased activity, fewer mutual encounters and larger interindividual distances, all of which may entail severe fitness costs. In female choice/male competition, socially deprived males mated earlier than socially enriched males; in male choice/female competition, socially deprived females were more likely to mate than socially enriched females. In neither mate choice situation did mating duration or body size at maturity differ between socially deprived and enriched mating opponents. Social isolation-induced shifts in mating behaviour may be interpreted as increased attractiveness or competitiveness or, more likely, as hastiness and reduced ability to assess mate quality. Overall, many of the social isolation-induced behavioural changes in *P. persimilis* are analogous to those observed in other animals such as cockroaches, fruit flies, fishes or rodents. We argue that, due to their profound and persistent effects, early social deprivation or enrichment may be important determinants in shaping animal personalities.

The social environment experienced early in life has profound influences on developmental, physiological and behavioural trajectories (Monaghan, 2008; West, King, & White, 2003). A crucial variable is whether individuals can socially interact or not, and this is especially true for animals living in groups (Krause & Ruxton, 2002). Animals normally reared together, or living in groups, are adapted to experiencing and interacting with conspecific individuals after birth or hatching. For such animals, social isolation (i.e. deprivation of social contact) is a highly stressful circumstance, with potentially severe and persistent adverse consequences (Cacioppo & Hawkley, 2009; Fuller, 1967).

The effects of social isolation (deprivation) on interrelated physiological, life history and behavioural traits have been extensively studied in vertebrates including humans (e.g. Blanchard, McKittrick, & Blanchard, 2001; Cacioppo & Hawkley, 2009; Gluck & Harlow, 1971), also because of their enormous importance to health (House, 2001; Scotti, Carlton, Demas, & Grippo, 2015). The occurrence of early social environment effects is widespread across animal taxa, but their expression varies with taxon-specific biology and ecology. Commonly affected traits are somatic growth and development, longevity, cognitive development, hormonal balance and social behaviour. For example, cichlids reared in isolation grow more slowly, shoal less, have impaired learning abilities, and are more aggressive and less cooperative in antipredator behaviours than cichlids reared in groups (Brandão, Braithwaite, & Goncalves-de-Freitas, 2015; Hesse, Anaya-Rojas, Frommen, & Thünken, 2015). In zebrafish, persistent social isolation decreases brain serotonin levels, which is a widespread effect of social isolation across vertebrates, and anxiety-related behaviours (Shams, Chatterjee, & Gerlai, 2015). The opposite occurs in chickens, in which social isolation increases anxiety (Suarez & Gallup, 1983). In mammals, social isolation commonly enhances defensiveness in subordinate individuals yet increases aggression in dominant ones (Blanchard et al., 2001). European rabbits reared in isolation suffer from reduced immune functions compared to those reared in groups (Rödel & Starkloff, 2014). Social isolation impairs white matter development and leads to abnormal connectomes in the brain cortex of adolescent rodents (Buwalda, Geerdink, Vidal, & Koolhaas, 2011; Liu et al., 2016). In nonhuman primates, social isolation early in life may impair sociability, decrease the ability to cope with stress, increase aggressiveness and/or lead to deficiencies in sexual and parental behaviours (for review, see Olsson & Westlund, 2007).

Analogous effects to those observed in vertebrates may occur in invertebrates. However, in comparison to vertebrates, social isolation in invertebrates is less extensively studied and less well understood, which particularly applies to behavioural effects. Most reports on social isolation (deprivation) in invertebrates deal with the effects on physiology and life history traits such as development or survival. For example, socially isolated ants have shorter life spans than those living in groups (Boulay, Quagebeur, Godzinska, & Lenoir, 1999; Koto, Mersch, Hollis, & Keller, 2015). Similarly, social isolation shortens longevity of *Drosophila* (Ruan & Wu, 2008). Mushroom bodies, which are important structures in insect brains for processing chemosensory inputs, of honeybees isolated early in life grow more slowly than those of bees reared in groups (Maleszka, Barron, Helliwell, & Maleszka, 2009). Socially deprived cockroaches develop more slowly, produce their oothecae later, are less exploratory, forage less and are less able to assess the quality of potential mates than those reared in groups (Lihoreau & Rivault, 2008; Lihoreau, Brepson, & Rivault, 2009; Woodhead & Paulson, 1983). Female cactus bugs reared in groups have a greater tendency to forage communally than those reared in isolation (Miller, Fletcher, Anderson, & Nguyen, 2012). Socially deprived male crickets are more aggressive towards females than socially experienced males (Kuriwada, 2016).

Here, we investigated the effects of complete social isolation early in life on development, body size at maturity, grouping behaviour and mate choice of the group-living plant-inhabiting predatory mite *Phytoseiulus persimilis*. *Phytoseiulus persimilis* is specialized to forage on spider mites of the family Tetranychidae. Group-living in *P. persimilis* is brought about by the patchy distribution of its prey and mutual attraction (Muleta & Schausberger, 2013; Sabelis, 1985; Strodl & Schausberger, 2012a, 2012b; Zhang & Sanderson, 1992). Gravid predatory mite females deposit their eggs inside the webbed patches/colonies of the spider mites, and the developing predator offspring, from larva to protonymph to deutonymph to adult, grow up together in their natal sites, provided that sufficient prey are available within the patch. The larvae are highly sensitive to environmental cues but do not need to feed to moult to the next stage, the protonymph, which is the first obligatory feeding stage (Strodl & Schausberger, 2012a, 2013). At 25 °C, total juvenile development from egg to adult takes about 7 days (Sabelis, 1985). Depending on the number and relatedness of the founder predator females, groups may consist of only kin or mixed kin and nonkin (Strodl & Schausberger, 2012a, 2013). If prey are scarce, offspring might find themselves in patches where they grow up alone, without having contact with conspecific individuals (Schausberger, 2004). Individuals reared in isolation are aggressive sibling cannibals, whereas those reared in a group avoid cannibalizing familiar individuals (Schausberger, 2004, 2007). Apart from cannibalism, the effects of early social isolation on interrelated life history and behavioural traits of *P. persimilis* are untested.

We hypothesized that social isolation early in life negatively affects early life history traits such as juvenile development and growth of *P. persimilis*, and makes them less sociable in grouping behaviour and less choosy in mate choice. We pursued these hypotheses in three separate experiments, in which we compared the behaviour of predatory mites having been isolated either in the larval and early protonymphal stage or throughout development and those reared in a group.

## Methods

### Predatory Mite Origin and Rearing

*Phytoseiulus persimilis* used in experiments were derived from a population originally collected in Greece. In the laboratory, the predators were maintained in piles of detached bean leaves infested by two-spotted spider mites, *Tetranychus urticae*, placed on acrylic tiles (15 × 15 × 0.5 cm). Three times per week, spider mite-infested bean leaves were added onto the tiles, which rested on water-soaked foam cubes (15 × 15 × 5 cm) inside plastic trays (20 × 20 × 6 cm) half-filled with tap water. Strips of moist tissue paper were wrapped around the edges of the tile to prevent the predators and their prey from leaving the arena. *Tetranychus urticae* was reared on whole common bean plants, *Phaseolus vulgaris*. To obtain *P. persimilis* eggs for experiments, about 20–40 gravid females, recognizable by their expanded bodies, were randomly withdrawn from the stock population and placed on a detached leaf arena, harbouring spider mites as prey, for oviposition. Detached leaf arenas consisted of a trifoliate leaf placed upside down on a water-soaked foam cube (5 × 5 × 5 cm) inside a plastic box (10 × 10 × 6 cm), with wet tissue wrapped around the edges of the leaf. No ethical approval or specific permit was needed for rearing and experimental use of *P. persimilis* and *T. urticae*, which are neither protected nor endangered species.

### Development (Experiment 1)

Predator eggs, <20 h old, were collected from oviposition arenas, with half of them singly placed into acrylic cages (subsequently dubbed ‘isolated’), each supplied with 8 spider mite eggs, and the other half placed in groups of three into acrylic cages (subsequently dubbed ‘grouped’), each supplied with 24 spider mite eggs. Each acrylic cage consisted of a circular cavity (diameter = 15 mm) laser-cut into an acrylic plate, closed at the bottom by gauze and on the upper side by a removable microscope slide (Schausberger, 1997). The cages were monitored twice per day in 8 and 16 h intervals for determining the developmental state of the predators. As soon as the predatory mites had reached the protonymphal stage, each individual (*N* = 37 for grouped and *N* = 36 for isolated) was removed from its cage and singly placed into a new cage, equipped with 12 spider mite eggs as prey, until reaching adulthood. When the mites had reached adulthood, their sex was determined. Each acrylic cage was meticulously cleansed with 75% ethanol, using cotton buds, before the experiment and was used only once in the experiment. Acrylic cages were kept at 25 ± 1 °C, 60 ± 5% relative humidity (RH) and a 16:8 h light:dark cycle.

### Mating Behaviour and Body Size (Experiment 2)

Predator eggs, <48 h old, were collected from the oviposition arenas, and half of them singly placed on detached leaf arenas, each harbouring two ovipositing spider mite females, to generate isolated predators, and the other half placed in groups of three on arenas, each harbouring four spider mite females, to generate grouped predators. Eggs produced by the spider mite females served as prey for the predators. The adult spider mite females were removed after 2–4 days, depending on the number of eggs produced by them. Isolated predators remained on their arena until reaching adulthood, with spider mite prey replenished as needed. Predators from the grouped treatment were removed from their arena when they had reached the deutonymphal stage, to prevent mating, and singly placed on new leaf arenas harbouring mixed spider mite stages as prey. When the predators had reached adulthood, females and males from the isolation and grouped treatments were marked with different water colour (Jolly; Brevillier Urban & Sachs, Vienna, AT) dots (red and black, randomly assigned), using a red marten-hair brush (size 0), on their dorsal shields (Schausberger, 2007; Strodl & Schausberger, 2012a, 2012b, 2013). To start the female (*N* = 42) and male (*N* = 52) choice tests, triplets consisting of one virgin female and two males (one from the isolation treatment and one from the grouped treatment), or of one male and two virgin females (one from the isolation treatment and one from the grouped treatment), respectively, were placed together on a leaf arena harbouring spider mites. Each triplet was repeatedly observed in predetermined intervals, after 10–20 min and then every 30 min for at least 3 h, to record the first chosen mate, time until mating occurred (mating latency) and mating duration. Each male and female predatory mite and each leaf arena was used only once in the experiment.

For body size measurements, isolated and grouped females (*N* = 77) and males (*N* = 42) used in the mate choice experiment were mounted in a drop of lactic acid on a microscope slide after the experiment. After storing the slides for 24 h at ~25 °C for clearing, the length of the dorsal shields was measured under the microscope. Dorsal shield length is a suitable indicator of body size in phytoseiid mites (Croft, Luh, & Schausberger, 1999).

### Grouping Behaviour (Experiment 3)

To obtain ‘isolated’, ‘grouped familiar’ and ‘grouped unfamiliar’ protonymphs for the experiment, we collected eggs from the predator oviposition arenas and placed them either singly, giving rise to isolated individuals, or in groups of 5–10 individuals, giving rise to grouped individuals, inside acrylic cages (dubbed ‘rearing cages’). As soon as the predators had reached the protonymphal stage inside the rearing cages, they were uniquely coloured (Jolly water colours) on their dorsal shields and ready for use in the experiment. Mites forming the subgroup ‘isolated’ on the experimental arena were coloured dark blue, light blue and violet; mites forming the subgroup ‘grouped familiar’ on the experimental arena were coloured dark green, light green and black; mites forming the subgroup ‘grouped unfamiliar’ were coloured dark brown, orange and red. Colour assignment was random and based on pilot experiments, verifying that different colours and sets of three colours, respectively, did not bias the behavioural tendencies of the predatory mites. The subgroup ‘isolated’ consisted of three protonymphs that had been reared in isolation in the rearing cage; the subgroup ‘grouped familiar’ consisted of three protonymphs that had been reared in the same rearing cage and were hence familiar with each other; the subgroup ‘grouped unfamiliar’ consisted of three protonymphs that had been raised with other larvae/protonymphs in three different rearing cages, and thus had been reared in groups but were unfamiliar with each other.

To start the experiment, we placed nine predatory mites, composed of three individuals reared in isolation (‘isolated’), three individuals reared together (‘grouped familiar’) and three individuals reared in three different groups (‘grouped unfamiliar’), on each experimental arena. Each experimental arena consisted of a circular white polypropylene disc (diameter = 14 mm) floating on the surface of a water column in an acrylic cylinder (height 2 cm, inner diameter 1.6 cm), leaving a 1 mm wide water film gap between the inner margin of the cylinder and the edge of the disc, impeding mite escaping. We randomly distributed 10–20 spider mite eggs on the arena to serve as prey for the predators during the experiment. Each experimental mite and each experimental arena was used only once in the experiment. Videotaping started after ~10 min of acclimatization following placement of the nine predatory mites on the arena. For videotaping, we used a Leica IC-A video module integrated in a Leica M50 microscope. To digitize the videos in the computer, the video module was interfaced by a frame grabber (HaSoTec FG-33-II; Indeo codec). Only replicates in which all nine protonymphs stayed for at least 25 min on the arena were subjected to visual analysis (*N* = 17). Mite activity (moving: yes/no) was assessed every 2.5 min; using stills, we measured interindividual distances every 5 min. For distance measurements, we used only data of stationary (i.e. nonmoving) mites. Whole videos were watched to record the encounter frequency of individuals within the ‘isolated’, ‘grouped familiar’ and ‘grouped unfamiliar’ subgroups. Encounters were scored when two individuals of the same subgroup came into mutual touching distance.

### Statistical Analyses

IBM SPSS Statistics for Windows, Version 23.0 (IBM Corp.; Armonk, NY, U.S.A.) was used for all statistical analyses. All tests were two tailed.

In experiment 1, we used separate generalized linear models (GLM; linear, identity link) to compare the developmental time of each life stage (egg, larva, protonymph, deutonymph) and total developmental time of predatory mites isolated or grouped during the larval and early protonymphal stage.

In experiment 2, we analysed mate preference of single females and males from the isolation or grouped treatment when given a choice between two potential mating partners, one reared in isolation and one reared in a group, by GLM (binomial, probit link). We compared first mating latency and first mating duration between mating partners that had been reared in isolation versus in a group by GLM (linear, identity ink). Similarly, we used GLM (linear, identity link) to compare the body length of male and female predatory mites reared in isolation versus in a group.

In experiment 3, we compared the interindividual distances within each subgroup of ‘isolated’, ‘grouped familiar’ and ‘grouped unfamiliar’ individuals (normal distribution, identity link) over time (used as nested inner subject variable) among subgroups by generalized estimating equations (GEE; Hardin & Hilbe, 2012). Least significant difference (LSD) tests were used for post hoc pairwise comparisons. We compared the numbers of encounters within each subgroup (isolated, grouped familiar, grouped unfamiliar individuals; binomial, logit link, counts of events) among subgroups by GLM. Encounters were aggregated into one value for each subgroup/video before analysis. Šidák tests were used for posthoc pairwise comparisons. Activity (moving: yes/no) within each subgroup (isolated, grouped familiar, grouped unfamiliar) was first aggregated into one value/video (binomial, logit, counts of events) and then compared among subgroups by GLM. Šidák tests were used for post hoc pairwise comparisons.

## Results

### Development (Experiment 1)

**Figure 1:**
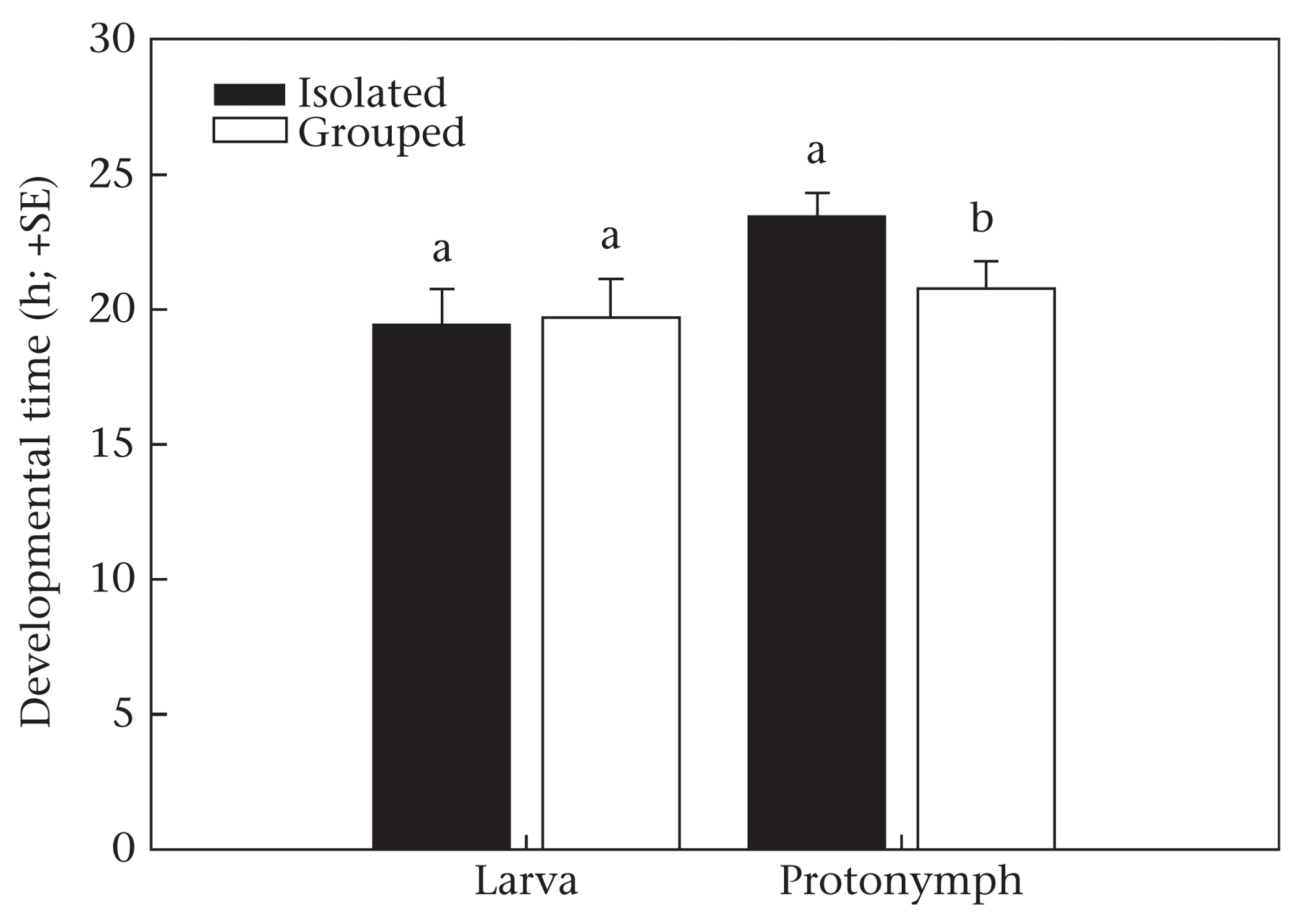
Development duration of larvae and protonymphs of *P. persimilis* that were either socially deprived (reared in isolation), or socially enriched (reared with conspecifics) during the larval and early protonymphal stage (experiment 1). Different letters above bars indicate significant differences between isolated and grouped individuals within life stages (GLM: *P* < 0.05).

Protonymphs (GLM: Wald χ^2^_1_=4.092, *P* = 0.04) but not larvae (Wald χ^2^_1_= 0.032, *P* = 0.86) reared in isolation developed more slowly than protonymphs and larvae reared in a group (Fig. 1). Deutonymph development duration (mean ± SE: grouped: 23.08 ± 0.94 min; isolated: 22.33 ± 1.05 min; Wald χ^2^_1_= 0.281, *P* = 0.60) and total development duration (grouped: 110.81 ± 1.65 min; isolated: 111.22 ± 1.21 min; Wald χ^2^_1_= 0.001, *P* = 0.99) were unaffected by having been isolated or grouped in the larval and early protonymphal stage.

### Mating Behaviour and Body Size (Experiment 2)

**Figure 2:**
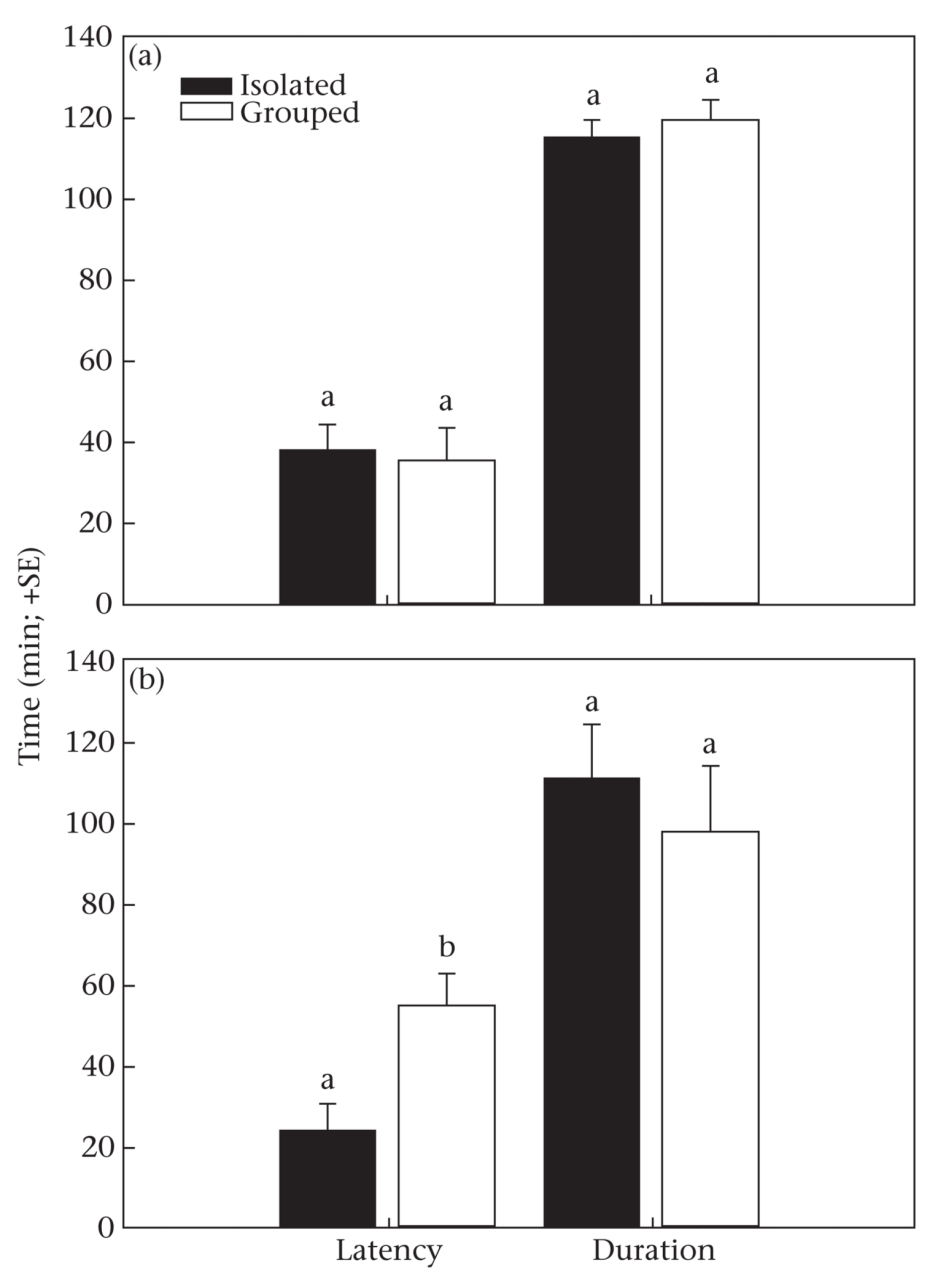
Latency and duration of the first mating in triplets consisting of (a) one male and two females or (b) one female and two males. In both (a) and (b), one individual of the competing sex was socially deprived (reared in isolation until adult) and the other was socially enriched (reared in a conspecific group) (experiment 2). Different letters the males above bars indicate significant differences between isolated and grouped individuals (GLM: *P* < 0.05).

Male rearing history (isolated or grouped) did not influence male choice (Wald χ^2^_1_ = 0.006, *P* = 0.94); both types of males were more likely to mate with females reared in isolation (33 of 52) than with those reared in a group (Wald χ^2^_1_ = 3.754, *P* = 0.05). Neither mating latency nor mating duration were influenced by the rearing history of the males (latency: Wald χ^2^_1_ = 0.677, *P* = 0.41; duration: Wald χ^2^_1_ = 0.049, *P* = 0.82) or that of the females (latency: Wald χ^2^_1_ = 0.045, *P* = 0.83; duration: Wald χ^2^_1_ = 0.378, *P* = 0.54) or the interaction between male and female rearing history (latency; Wald χ^2^_1_ = 0.909, *P* = 0.34; duration: Wald χ^2^_1_ = 0.885, *P* = 0.35; Fig. 2a). Females, regardless of their rearing history (Wald χ^2^_1_ = 0.727, *P* = 0.39), were equally likely to mate with males reared in isolation (N = 25) and males reared in a group (N = 17) (Wald χ^2^_1_ = 2.073, *P* = 0.15). However, mating latency was shorter with males reared in isolation than with males reared in a group (Wald χ^2^_1_ = 4.704, *P* = 0.03), regardless of female rearing history (Wald χ^2^_1_ = 0.004, *P* = 0.949) and the interaction between male and female rearing history (Wald χ^2^_1_ = 1.377, *P* = 0.241). Mating duration did not differ between males reared in isolation and males reared in a group (Wald χ^2^_1_ = 0.035, *P* = 0.85), and was neither influenced by female rearing history (Wald χ^2^_1_ = 1.155, *P* = 0.28) nor by the interaction between male and female rearing history (Wald χ^2^_1_ = 1.637, *P* = 0.20; Fig. 2b).

**Figure 3:**
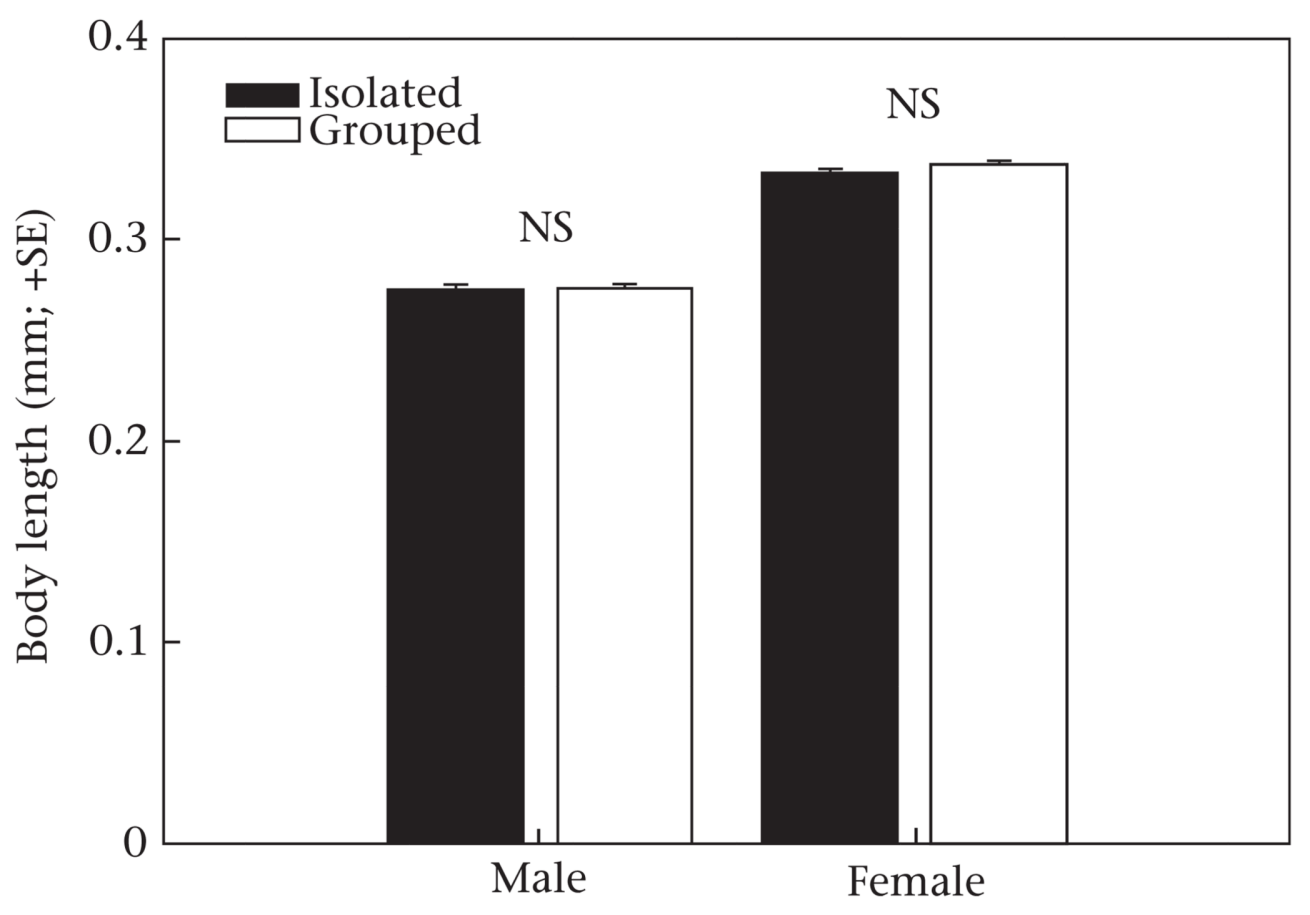
Body length at maturity of male and female *P. persimilis* that had been socially deprived (reared in isolation until adult) or socially enriched (reared in a conspecific group) (experiment 2). GLM: *P* > 0.05.

Females had larger bodies than males (GLM: Wald χ^2^_1_ = 907.423, *P* < 0.001), but neither male nor female body size (Wald χ^2^_1_ = 1.002, *P* = 0.32) nor the interaction between sex and rearing history (Wald χ^2^_1_ = 1.098, *P* = 0.30) differed between individuals reared in isolation and individuals reared in a group (Fig. 3).

### Grouping Behaviour (Experiment 3)

**Figure 4:**
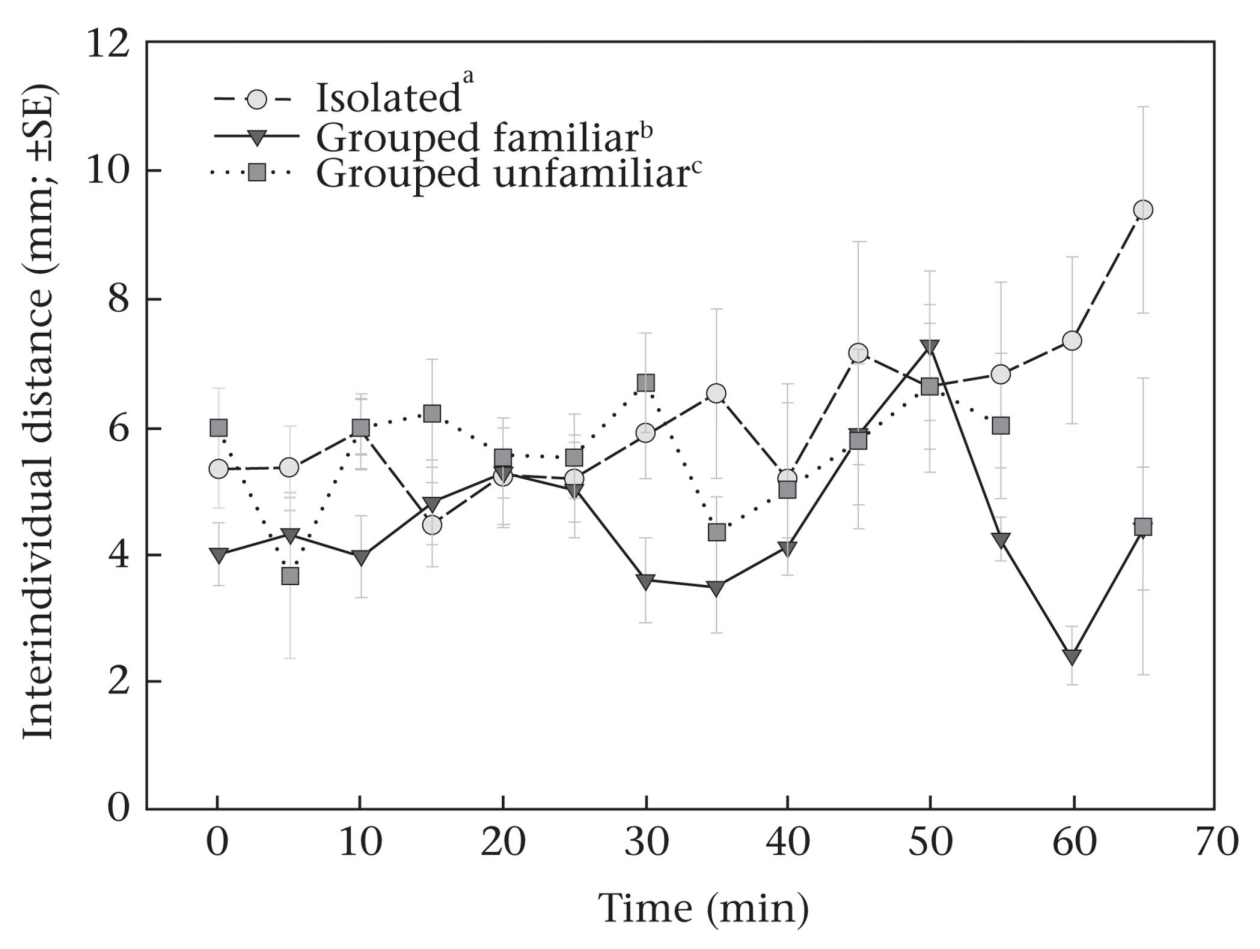
Time-dependent interindividual distances within each of three subgroups of a group of nine protonymphs of *P. persimilis* (experiment 3). Before the experiment, all three individuals of a subgroup had experienced the same social conditions in the larval and early protonymphal stage: reared in social isolation (‘isolated’), reared together (‘grouped familiar’), or reared in three different groups (‘grouped unfamiliar’). Different superscript letters accompanying subgroup designations indicate significant differences between ‘isolated’, ‘grouped familiar’ and ‘grouped unfamiliar’ individuals (GEE: subgroup as main effect: *P* < 0.05; subgroup nested in time: *P* < 0.001).

**Figure 5:**
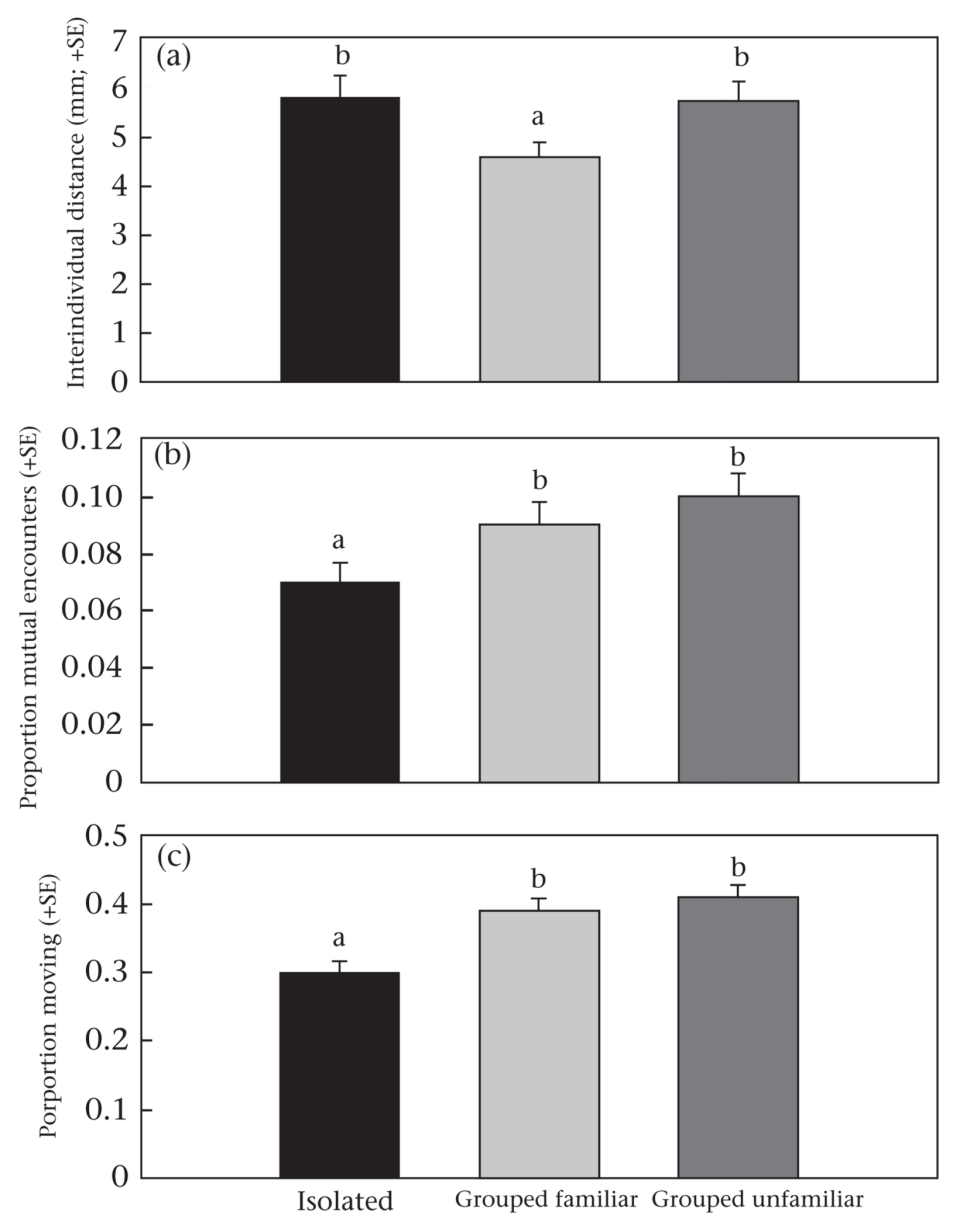
(a) Interindividual distances, (b) mutual encounters and (c) activity within each of three subgroups of a group of nine protonymphs of *P. persimilis* (experiment 3). Before the experiment, all three individuals of a subgroup had experienced the same social conditions in the larval and early protonymphal stage: reared in social isolation (‘isolated’), reared together (‘grouped familiar’), or reared in three different groups (‘grouped unfamiliar’). Different letters above bars indicate significant differences between ‘isolated’, ‘grouped familiar’ and ‘grouped unfamiliar’ individuals (GLM: *P* < 0.05).

The interindividual distances among ‘grouped familiar’ protonymphs were generally shorter than those among ‘grouped unfamiliar’ and among ‘isolated’ protonymphs (GEE: Wald χ^2^_2_ = 7.617, *P* = 0.02; Figs 4, 5a). The interindividual distances also developed differently over time (Wald χ^2^_38_ = 884.085, *P* < 0.001). While the distances between ‘isolated’ individuals increased over time, those between ‘grouped familiar’ and ‘grouped unfamiliar’ individuals stayed at about the same levels (Fig. 4). ‘Grouped familiar’ and ‘grouped unfamiliar’ protonymphs encountered each other more often than did ‘isolated’ individuals (GLM: Wald χ^2^_2_ = 6.933, *P* = 0.03; Fig. 5b). ‘Isolated’ individuals moved less than ‘grouped familiar’ and ‘grouped unfamiliar’ individuals (GLM: Wald χ^2^_2_ = 19.792, *P* < 0.001; Fig. 5c).

## Discussion

Our study documents that the social conditions experienced early in life have profound consequences for life history and behavioural trajectories of the predatory mite *P. persimilis*. Social deprivation (i.e. reared in isolation) retarded development, decreased activity, decreased intragroup sociability and altered mating behaviour, as compared to social enrichment (i.e. reared with conspecific individuals). In grouping and mating behaviour, individuals reared in isolation seemed to be less socially competent, with social competence defined as ‘the ability of an animal to optimise the expression of its social behaviour as a function of the available social information’ (Taborsky & Oliveira, 2012, p. 679), as compared to those reared in a socially enriched environment. Many effects of social isolation observed in *P. persimilis* are analogous to those observed in other animals, both vertebrates and invertebrates, such as mammals, fishes, birds and insects (Blanchard et al., 2001; Buwalda et al., 2011; Lihoreau et al., 2009; Olsson & Westlund, 2007; Sachser, Hennessy, & Kaiser, 2011).

Social isolation has been observed to induce retarded development or slower growth, among others, in fishes (Hesse et al., 2015) and cockroaches (Lihoreau & Rivault, 2008; Woodhead & Paulson, 1983). In cockroaches, social isolation affects a suite of coupled behavioural traits, dubbed a ‘behavioural syndrome’ (Sih, Bell, & Johnson, 2004), including decreased exploratory and foraging activities (Lihoreau & Rivault, 2008; Lihoreau et al., 2009). These traits were similarly coupled and affected by early social experience in *P. persimilis*. Socially deprived individuals developed more slowly, were less active, which might be indicative of reduced exploration and foraging, and were less sociable in grouping behaviour, as compared to socially enriched individuals. Socially deprived individuals also encountered each other less often and kept larger interindividual distances within groups than socially enriched individuals. Similarly, social isolation reduced positive thigmotaxis in zebrafish (Shams et al., 2015) and cockroaches (Lihoreau & Rivault, 2008). Strikingly, intragroup activity and frequency of mutual encounter were only determined by social deprivation/enrichment early in life but not by familiarity, that is, these traits did not differ between ‘grouped familiar’ and ‘grouped unfamiliar’ individuals. In contrast, the interindividual distances were determined by familiarity and were shorter in familiar than unfamiliar individuals, regardless of whether the unfamiliar individuals had been socially isolated or enriched. The latter finding is in accordance with previous studies, in which we observed, for both immature and adult *P. persimilis*, that familiar individuals stay closer together than unfamiliar ones (Strodl & Schausberger, 2012a, 2012b, 2013). Furthermore, this finding suggests context dependency of the effects of early social enrichment. Individuals that had been socially enriched in exactly the same way (grouped with conspecifics), behaved, later in life, differently towards ‘grouped familiar’ and ‘grouped unfamiliar’ individuals.

The mating behaviour of *P. persimilis* is characterized by males actively searching for females (Amano & Chant, 1978), but after mate finding, the female controlling whether copulation occurs (Walzer & Schausberger, 2015). Accordingly, in the male choice situation, higher mating likelihood with socially isolated females may be interpreted as social isolation (1) decreasing female choosiness (females accept any approaching male and mate more readily than socially enriched females do), or (2) increasing female competitiveness and willingness to mate, or (3) enhancing female defensiveness and thus attractiveness to males. Option (3) could simply represent an epiphenomenon of the females being less active and thus being easier for males to locate. In the female choice situation, shorter mating latency with socially isolated males may be interpreted as social isolation (1) decreasing the choosiness of males, which quickly approached the females, or (2) increasing male attractiveness to the females, accepting them quickly for mating, or (3) increasing male competitiveness, allowing them to outpace their rivals. Option (3) is unlikely because, in the grouping behaviour context, socially isolated individuals were less active than socially enriched ones. In general, the social environment experienced early in life could be indicative of the intensity of future mate competition (Bretman, Westmancoat, Gage, & Chapman, 2013) and shape male behaviour accordingly. For example, Hesse, Bakker, Baldauf, and Thünken (2016) observed reduced interest towards potential mates and reduced intrasexual aggression in both sexes of socially isolated cichlids, as compared to enriched ones. In contrast, McGhee and Travis (2011) observed increased aggression by isolated male killifish towards females. Overall, in fishes, the effects of social isolation on aggression in mating behaviour seem strongly species and context dependent, as indicated by the lack of general trends (Gomez-Laplaza & Morgan, 2000). In *P. persimilis*, the sex ratio is usually strongly female biased (Amano & Chant, 1978) yet only a male-biased early social environment would be indicative of intense future mate competition, possibly resulting in shorter mating latencies of grouped than isolated males. In our experiments, the opposite, shorter mating latencies of isolated than grouped males, was the case, which is expected when grouped males experience a female-biased early social environment. Female-biased early social environments indicate weak future mate competition and should thus result in choosy males that take time to select their mating partners. In the female choice situation, shorter mating latencies did not correlate with higher mating likelihood, and were thus not indicative of mate preference, whereas short mating latencies are indicative of mate preferences in no-choice situations (P. Schausberger, personal observation). We thus favour the interpretation that social isolation induces hasty behaviour and/or decreases the ability to assess the quality of potential mating partners in both sexes. A similar phenomenon was observed in cockroaches (Lihoreau et al., 2009).

Whether or not the behavioural changes brought about by social isolation are nonadaptive or maladaptive, or even adaptive, depends on the species, the affected trait and the conditions of the future social environments (e.g. Gomez-Laplaza & Morgan, 2000; Sachser et al., 2011). In the case of *P. persimilis*, slower development delays, and thus negatively affects, body size-dependent access to resources such as food and dispersal from depleted prey patches, and is thus nonadaptive. Social isolation-induced changes in grouping behaviour (i.e. less associating with other individuals, fewer mutual encounters and larger interindividual distances) are largely disadvantageous. Previous studies have shown that actively searching for, and grouping with, familiar individuals improves foraging (Strodl & Schausberger, 2012a) and enhances prime fitness traits, such as the reproductive rate (Strodl & Schausberger, 2013) and survival chance under predation risk (Strodl & Schausberger, 2012b). The adaptive value of the changed behavioural traits in the mate choice experiment, shorter mating latencies of isolated females in male choice and higher mating likelihood of isolated males in female choice, is difficult to predict and dependent on how those changes are interpreted. If interpreted as increased attractiveness, those changes would be considered fitness enhancing. However, for the above described reasons we favour the interpretation of a decreased ability to assess mate quality or hasty behaviour; if so, the social isolation-induced changes in mating behaviour would be considered detrimental to fitness.

Our study underlines the suitability of the predatory mite *P. persimilis* as a model arthropod species in animal behaviour and ecology, and adds this species to the list of model animals, such as macaques, rodents, fishes, cockroaches and fruit flies, used to scrutinize the effects of social stress during development on interrelated behavioural, physiological and life history traits (Blanchard et al., 2001; Buwalda et al., 2011; Olsson & Westlund, 2007; Sachser et al., 2011). Some affected traits, especially grouping behaviour in the interplay with familiarity, have high fitness relevance. If traits change consistently and persistently, the social setting early in life (isolation versus enrichment) might be an important, but largely neglected, determinant of the formation of animal personalities (McGhee & Travis, 2011; Stamps & Groothuis, 2010).
